# Exosomes from tendon derived stem cells promote tendon repair through miR-144-3p-regulated tenocyte proliferation and migration

**DOI:** 10.1186/s13287-022-02723-4

**Published:** 2022-02-23

**Authors:** Kai Song, Tao Jiang, Pin Pan, Yao Yao, Qing Jiang

**Affiliations:** 1 State Key Laboratory of Pharmaceutical Biotechnology, Division of Sports Medicine and Adult Reconstructive Surgery, Department of Orthopedic Surgery, Nanjing Drum Tower Hospital, The Affiliated Hospital of Nanjing University Medical School, 321 Zhongshan Road, Nanjing, 210008 Jiangsu People’s Republic of China; 2 Laboratory for Bone and Joint Disease, Model Animal Research Center (MARC), Nanjing University, Nanjing, 210093 Jiangsu People’s Republic of China; 3 Department of Orthopedic Surgery, The Affiliated Yixing Hospital of Jiangsu University, Wuxi, 214200 Jiangsu People’s Republic of China; 4 Department of Orthopedic Surgery, The Second People’s Hospital of Hefei, Hefei, 230011 Anhui People’s Republic of China

**Keywords:** Tendon repair, Tendon derived stem cells, Exosomes, miR-144-3p, ARID1A

## Abstract

**Background:**

Tendon derived stem cells (TDSCs) have proven to be effective in tendon repair by secreting paracrine factors, which modulate the function of resident cells and inflammatory process. Exosomes, which are secreted from cells to mediate intercellular communication, may be used to treat tendon injuries. Here, we aimed to determine the effects of exosomes from TDSCs (TDSC-Exos) on tendon repair and to explore the underlying mechanism by investigating the role of microRNAs (miRNAs).

**Methods:**

TDSC-Exos were isolated from TDSC conditioned medium. In vitro studies were performed to investigate the effects of TDSC-Exos on the proliferation, migration, cytoprotection, collagen production and tendon-specific markers expression in tenocytes. In order to determine the therapeutic effects of TDSC-Exos in vivo, we used a scaffold of photopolymerizable hyaluronic acid (p-HA) loaded with TDSC-Exos (pHA-TDSC-Exos) to treat tendon defects in the rat model. Subsequently, RNA sequencing and bioinformatic analyses were used to screen for enriched miRNAs in TDSC-Exos and predict target genes. The miRNA-target transcript interaction was confirmed by a dual-luciferase reporter assay system. In order to determine the role of candidate miRNA and its target gene in TDSC-Exos-regulated tendon repair, miRNA mimic and inhibitor were transfected into tenocytes to evaluate cell proliferation and migration.

**Results:**

Treatment with TDSC-Exos promoted proliferation, migration, type I collagen production and tendon-specific markers expression in tenocytes, and also protected tenocytes from oxidative stress and serum deprivation. The scaffold of pHA-TDSC-Exos could sever as a sustained release system to treat the rat model of tendon defects. In vivo study showed that TDSC-Exos promoted early healing of injured tendons. Rats treated with TDSC-Exos had better fiber arrangement and histological scores at the injury site. Besides, the injured tendons treated with TDSC-Exos had better performance in the biomechanical testing. Therefore, the pHA-TDSC-Exos scaffold proved to facilitate tendon repair in the rat model. miR-144-3p was enriched in TDSC-Exos and promoted tenocyte proliferation and migration via targeting AT-rich interactive domain 1A (ARID1A).

**Conclusions:**

TDSC-Exos enhanced tenon repair through miR-144-3p-regulated tenocyte proliferation and migration. These results suggest that TDSC-Exos can serve as a promising strategy to treat tendon injuries.

**Supplementary Information:**

The online version contains supplementary material available at 10.1186/s13287-022-02723-4.

## Background

Tendon pathologies, usually classified as chronic or acute injuries, represent one of the most common musculoskeletal disorders worldwide [1]. Chronic tendon injury, related to overuse and characterized by pain and impaired function, decrease quality of life in the individual and increase economic burden to society. The current treatments for chronic tendon injury range from physiotherapy, nonsteroidal anti-inflammatory drugs to local injection of glucocorticoid. Tendon rupture can be caused by acute or chronic injury and often requires surgery. However, the outcomes of these treatments are not satisfactory, since they are not able to completely restore the injured tendon to its native condition.

Cell-based therapies have been increasingly employed to repair tendon injuries during the last two decades. Cells derived from different sources have proven to be effective, including tenocytes, dermal fibroblasts, bone marrow mesenchymal stem cells (BMSCs), adipose-derived stem cells (ADSCs), and embryonic stem cells (ESCs) [2–6]. A population of stem cells, which are termed tendon derived stem cells (TDSCs), has been identified in tendon tissues with universal stem cell characteristics, such as self-renewal, multilineage differentiation and clonogenicity [7]. Allogenic TDSCs have proven to suppress immunoreactions and promote tendon repair in a rat model [8]. Furthermore, TDSCs have greater tenogenesis, clonogenicity and proliferation capacity compared with BMSCs [9], which indicates that TDSCs may represent a better cell source for tendon repair.

However, there are several potential risks associated with cell-based therapies, including pathogen transmission, immune rejection, and potential tumorigenesis. Technical complexity and legitimate concerns also increase the difficulty of therapeutic translation. Therefore, an alternative approach is warranted to achieve the desired effects and avoid the drawbacks of cell-based therapies.

The mechanism of cell-based therapies has long been considered to be the proliferation, differentiation and migration of transplanted cells. Recently, increasing evidence indicates that transplanted cells repair injured tissues by secreting paracrine factors, which modulate the function of resident cells and inflammatory process [10]. Therefore, we may solve the issues of cell-based therapies by finding a carrier, which contains paracrine factors released by transplanted cells and delivers them to the target site.

Exosomes, which have attracted increasing attention recently, may be able to serve as the carrier. Exosomes are membrane vesicles secreted by almost all cell types with a diameter of 30–150 nm. They contain abundant proteins, lipids, DNA, mRNA, and non-coding RNA, which modulate the function of recipient cells after uptake. Exosomes mediate intercellular communication and have proven to be able to repair injured tissues, such as myocardial ischemia/reperfusion injury [11], kidney injury [12], bone defect [13], and osteoarthritis [14]. Exosomes derived from BMSCs and ADSCs have also been reported to promote tendon healing in rat models [15–17]. Modified umbilical cord stem cell-derived exosomes could inhibit tendon adhesion and enhance tendon regeneration [18, 19]. Since TDSCs have abovementioned advantages in tendon repair, we used exosomes derived from TDSCs in this study. Besides, there was no data about the microRNA (miRNA) expression profiling of TDSC-derived exosomes (TDSC-Exos). Therefore, we performed miRNA sequencing and explored the role of exosomal miRNA in tendon repair.

We hypothesized that exosomes secreted by TDSCs could enhance tendon repair. In the present study, we explored the effects of TDSC-Exos on tenocytes in vitro. Subsequently, a rat model of tendon defects was employed to determine the therapeutic effectiveness of TDSC-Exos. In order to reveal the underlying mechanism, we conducted miRNA sequencing and found that miR-144-3p was enriched in TDSC-Exos. Futher study regarding exosomal miR-144-3p determined its role in regulating the proliferation and migration of tenocytes via targeting AT-rich interactive domain 1A (ARID1A).

## Methods

### Ethics statement

This study was approved by the Institutional Ethics Committee of Drum Tower Hospital Affiliated to Medical School of Nanjing University. All animals were treated in accordance with the guidelines for the care and use of laboratory animals published by the National Institutes of Health (eighth edition).

### Isolation and characterization of TDSCs

Six-week-old male Sprague-Dawley (SD) rats weighting 200–250 g were provided by Nanjing Medical University Animal Centre. A total of 16 rats were used to isolate TDSCs. Tendons from 3–4 rats were mixed together to isolate cells every time. The procedure of TDSC isolation and culture was established by a previous study [20]. In brief, the patellar tendons were excised from healthy rats and washed in sterile phosphate-buffered saline solution (PBS) with 10% PSN (500 μg/mL penicillin, 500 μg/mL streptomycin, and 1000 μg/mL neomycin) for 5 min. Tendon tissues were minced and then digested with type I collagenase (3 mg/mL; Sigma-Aldrich, St Louis, MO, USA) and passed through a 70 μm cell strainer (Becton Dickinson, Franklin Lakes, NJ, USA) to yield single-cell suspension. The cells were washed with PBS twice and resuspend in low glucose Dulbecco’s Modified Eagle Medium (LG-DMEM) (Gibco, Invitrogen corporation, Carlsbad, USA) with 10% fetal bovine serum (FBS), 50 μg/mL penicillin, 50 μg/mL streptomycin, and 100 μg/mL neomycin. The isolated cells were plated at a low density (500 cells/cm^2^) and cultured at 37 °C in 5% CO_2_ to form colonies, and washed with PBS to remove the non-adherent cells at day 2. The cells were trypsinized and resuspend as passage 0 (P0) at day 7. The cells at passage 3 were used in subsequent experiments.

In order to determine the ability of colony formation, TDSCs were plated at 5, 50, 500, and 5000 cells/cm^2^ in 60 mm dishes and cultured for 10 days. The cells were stained with 0.5% crystal violet (Sigma, St. Louis, MO) to reveal the colonies. Multipotential differentiation of TDSCs was assessed by testing their osteogenenic, adipogenic and chondrogenic potential as described previously [20]. After culturing TDSCs in osteogenic, adipogenic and chondrogenic induction medium, we performed Alizarin Red S, Oil Red O and Safranin O staining. The expression of stem cell surface makers in TDSCs was analyzed by flow cytometry. Anti-CD31 (50-0310-80; eBioscience), anti-CD106 (200403; BioLegend), anti-CD44 (12-0444-80; eBioscience), and anti-CD90 (ab33694; Abcam) were the antibodies used in this study. The percentage of cells with positive signal was calculated using the FACScan program (Becton Dickinson, San Jose, CA).

### Isolation and characterization of TDSC-Exos

After reaching 70% confluency in 150 cm^2^ cell culture dishes, TDSCs were rinsed three times with PBS and then cultured in LG-DMEM medium with 10% FBS (exosomes free) for 48 h. The conditioned medium was collected for the exosome isolation. To remove cellular debris, the obtained medium was centrifuged at 300×*g* for 10 min, 2000×*g* for 10 min and 10,000*g* for 60 min at 4 °C. After centrifugation, the supernatant was filtered with a 0.22 μm filter (Merck–Millipore) to remove the microparticles. Subsequently, the supernatant was ultracentrifuged at 100,000×*g* for 70 min. Exosomes at the bottom of the centrifuge tube were resuspended in PBS, and then were centrifuged at 100,000×*g* for 1 h at 4 °C. In order to obtain purified exosomes, we removed the supernatant and added 200 μL of PBS to resuspend the pellet.

The size distribution of the exosomes was measured using Nanosight viewer (Malvern Instruments, Malvern, UK), and was analyzed using Zetasizer software (Malvern). The morphology of the exosomes was imaged by transmission electron microscopy (TEM). The exosomes were loaded onto copper grids coated with Formvar (Structure Probe, Inc., PA, USA). The grids were contrasted using 2% uranyl acetate, dried and then captured using a digital camera (Olympus®, Tokyo, Japan). The concentration of the protein in exosomes was used to represent the concentration of exosomes in this study. The exosomes were lysed in 50 μL of RIPA buffer and sonicated in 4 °C water bath. The protein content of the concentrated exosomes was determined using a bicinchoninic acid (BCA) protein assay kit (Thermo Scientific, USA) according to the manufacturer's instruction. Specific markers of exosomes (CD9, CD63, CD81, TSG101) were detected by western blotting.

### Isolation and culture of tenocytes

A total of nine rats were used to isolate tenocytes. Tendons from three rats were mixed together to isolate cells every time. The mid-substance tissue of the rat patellar tendon was transferred to a 100 mm culture dish containing PBS, and epitenon membrane sheet was then removed. The tendon was cut into small pieces (about 3 mm^3^). All pieces were placed in a 60 mm culture dish containing 2 mL LG-DMEM medium with 10% FBS, 50 μg/mL penicillin, 50 μg/mL streptomycin, and 100 μg/mL neomycin, and then incubated at 37 °C in 5% CO_2_. The medium was changed every three days. At day 10–14, tissue pieces were removed. The adherent cells were trypsinized and cultured as P0. Tenocytes from passage 3 were used for all experiments.

### The effects of TDSC-Exos on tenocytes in vitro

Before we study the effects of TDSC-Exos on tenocytes, the exosome uptake of tenocytes should be determined first. Exosomes were labeled with PKH67 (Sigma-Aldrich, St. Louis, MO, USA) as described previously [21]. Tenocytes were incubated with labeled exosomes for 6 h at 37 °C in 5% CO_2_. After incubation, the cells were washed twice with PBS and fixed in polyformaldehyde for 15 min and stained with DAPI for 5 min. The slide was mounted with ProLong Gold Antifade Reagents, and the internalization of the exosomes was observed by fluorescence microscopy (Leica Microsystems, Wetzlar, Germany).

The effects of the exosomes on the proliferation, anti-oxidant stress and anti-serum deprivation of tenocytes were assessed by cell counting kit-8 (CCK-8; Dojindo Molecular Technologies, Inc., Kumamoto, Japan) according to the manufacturer’s instruction. Briefly, tenocytes were seeded into 96-well plates at an initial density of 4 × 10^3^ cells per well. Tenocytes cultured in the medium with 200 μM of H_2_O_2_ served as the oxidative stress cellular model. The model of serum deprivation was established by using culture medium without FBS. Different concentrations of exosomes (1, 10, 50, 100 μg/mL) were added and incubated with tenocytes for 24 h. Subsequently, 180 μL of culture medium mixed with 20 μL of CCK-8 were added into each well and incubated for 1 h at 37 °C before measuring product concentration with a microplate reader at a wavelength of 450 nm. Multiple group comparison was conducted using one-way ANOVA analysis. Subsequently, the values of each group were compared with those of the control group using Dunnett’s multiple comparison test. TDSC-Exos at the concentration of 100 μg/mL were found to promote proliferation of tenocytes and protect tenocytes from oxidant stress and serum deprivation. Therefore, this concentration was used in the following in vitro studies.

Type I collagen produced by tenocytes after treatment with TDSC-Exos was evaluated by Sirius red staining. Tenocytes were cultured with exosomes for 10 days. Before performing Sirius red staining, cells were fixed with 70% ethanol for 30 min and washed 3 times. The deposited collagen was stained with 0.1% Sirius red in saturated aqueous solution of picric acid. Images were taken using microscope Leica DMIRB.

Real-time quantitative PCR (qRT-PCR) was conducted to assess the effects of TDSC-Exos (50 or 100 μg/mL) on the specific gene expression in tenocytes. The β-actin primers were 5′-ATCGTGGGCCGCCCTAGGCA-3′ (forward) and 5′-TGGCCTTAGGGTTCAGAGGGG-3′ (reverse). The Scleraxis (Scx) primers were 5′-AACACGGCCTTCACTGCGCTG-3′ (forward) and 5′-CAGTAGCACGTTGCCCAGGTG-3 (reverse). The Col1a1 primers were 5′-GGAGAGAGCATGACCGATGG-3′ (forward) and 5′-GGGACTTCTTGAGGTTGCCA-3′ (reverse). The Decorin (Dcn) primers were 5′-ATGATTGTCATAGAACTGGGC-3′ (forward) and 5′-TTGTTGTTATGAAGGTAGAC-3′ (reverse). Multiple group comparison was conducted using one-way ANOVA analysis. Subsequently, the values of each group were compared with those of the control group using Dunnett’s multiple comparison test. qRT-PCR was also performed in the following experiments to determine the level of expression of miR-144-3p and ARID1A. The Uracil6 (U6) primers were 5′-GCTTCGGCAGCACATATACTAAAAT-3′ (forward) and 5′-CGCTTCACGAATTTGCGTGTCAT-3′ (reverse). The miR-144-3p primers were 5′-GGCGTACAGTATAGATGAT-3′ (forward) and 5′-GAGCAGGCTGGAGAA-3′ (reverse). The ARID1A primers were 5′-TGCAGCTGCTGATTCCAAGA-3′ (forward) and 5′-ACTGAGTTGCTCCTCATGCC-3′ (reverse). U6 served as an internal microRNA control and β-actin severed as an internal mRNA control.

Transwell assay and scratch wound healing assay were performed to evaluated tenocyte migration after treatment with TDSC-Exos. Briefly, 5 × 10^4^ tenocytes were seeded into the upper chamber of a 24-well 8-μm-pore-size transwell plate (Corning, Corning, NY, USA). Then, 600 μL of medium containing exosomes at the concentration of 100 μg/mL was added into the lower chamber. After incubation for 24 h, cells were fixed with 4% paraformaldehyde and then stained for 5 min with 0.5% crystal violet. The cells on the upper surface of the membranes were removed with a cotton swab after washing three times in PBS. Five randomly-selected fields (100 × magnification) per well were photographed using a Leica microscope and assessed by two researchers in a blinded manner. The wound-healing assay was performed by scratching a confluent layer of tenocytes in a 24-well plate using a 20 μL pipette tip. The loose cells were removed by washing with PBS, and then 200 μL of medium with or without exosomes was added. Images of the wound-healing process were captured at 0 h, 24 h and 48 h.

### Preparation of photopolymerizable hyaluronic acid loaded with TDSC-Exos

An ultraviolet-reactive scaffold of photopolymerizable hyaluronic acid (p-HA) integrated with TDSC-Exos was prepared according to our previous study [22]. The scaffold contained 50 mg/mL of p-HA and 100 μg/mL of TDSC-Exos, which was used for the subsequent animal study.

In order to assess the distribution of TDSC-Exos in p-HA, exosomes were labeled with the fluorescent dye CM-Dil (Sigma-Aldrich, St. Louis, MO, USA). Subsequently, the pellet containing CM-Dil-labeled exosomes was resuspended in 100 μL p-HA solution. The mixture was dropped on the slide and mounted with ProLong Gold Antifade Reagents, and the distribution of the exosomes was observed using fluorescence microscopy (Leica Microsystems, Wetzlar, Germany).

Before we conducted in vivo study, the exosome retention ability of the scaffold was tested. The scaffold was prepared by 200 μL of p-HA and 2 × 10^12^ exosomes, and then was placed in a 48-well plate with 200 μL of PBS. The PBS was collected daily and replaced by 200 μL of fresh PBS. The quantity of released exosomes in the collected PBS were detected by Nanosight viewer platform as described above. The quantity of retained exosomes was calculated by the total amount of exosomes minus the quantity of released exosomes.

### The effects of TDSC-Exos on tendon repair in vivo

Seventy-two SD rats (7–8 weeks, body weight of 250–320 g) were used in this study. To create the model of tendon defect, the central one-third of the patellar tendon was removed as previously described [23]. Fifty-four operated rats were randomly divided into 3 groups (*n* = 6 in each group at each time point): control group, group with pHA treatment and group with pHA-TDSC-Exos treatment. Injury gourp without pHA or pHA-TDSC-Exos treatment served as control group. For pHA and pHA-TDSC-Exos group, pHA or pHA-TDSC-Exos solution was injected into the defect site, and hydrogel scaffold was formed via ultraviolet irradiation for 1 min. The wound was then closed in layers. Animals were allowed to have free-cage activity.

Rat patellar tendons were harvested at 2 week, 4 weeks and 8 weeks postoperatively to conduct histological analyses. The tissue sections were stained with H&E. Tendons harvested at 8 weeks after surgery were assessed by the modified Movin score system [24], which could semi-quantify the histological changes in the tendons. The histological score of a tendon tissue could vary between 0 (normal tendon) and 21 (the most severe abnormality detectable). One-way ANOVA with Tukey’s multiple comparison test was conducted to analyze the difference among these groups. Masson’s trichrome staining was performed to examine the general appearance of the collagen fibers in the tendons. Immunohistochemistry was subsequently conducted to evaluate the production of type III collagen during the process of tendon repair.

Tendons of another 6 rats from each group were used to conduct biomechanical tests using a universal mechanical testing machine (ElectroPlus E3000; Instron, MA, USA). The regenerated tissue in the window wound with the bony ends was isolated from the harvested patellar tendons at week 8. The cross-sectional area was measured by a laser micrometer (Model LS7030-MT; Keyence, Elmwood Park, NJ). The specimen was fixed onto the clamps of the Instron machine and preloaded to 0.1 N to remove slack. Subsequently, a constant displacement rate at 40 mm/min was applied until failure. The ultimate stress and Young’s modulus were obtained directly from the machine. One-way ANOVA with Tukey’s multiple comparison test was conducted to analyze the difference among these groups.

### miRNA library construction and sequencing

miRNA library preparation and sequencing were performed by a commercial service (Ribobio, China). Briefly, total RNAs were extracted from exosomes obtained from medium. Both 3′ and 5′ adaptors were added to each end, respectively, followed by reverse transcription and polymerase chain reaction (PCR) amplification. The PCR products ranging between 18 and 30 nucleotides (nt) were purified by electrophoresis and sequenced using the Illumina HiSeq 2500 platform.

### Bioinformatic analysis

According to the miRNA expression profiles of TDSC-Exos and tenocytes, the t test was employed to determine the differential miRNAs expression (fold change > 1.0; *p* < 0.05). Candidate target genes of the top 10 enriched miRNAs in TDSC-Exos were predicted by four databases (TargetScan, miRanda, miRWalk, and miRTarBase), and then STRING online database was employed to identify those protein–protein interactions (PPI) with a combined score > 0.9. Besides, gene ontology (GO) terms and Kyoto Encyclopedia of Genes and Genomes (KEGG) pathway enrichment analyses were performed to explore the most related processes and signaling pathways.

### Dual-luciferase reporter assay

A dual-luciferase reporter assay system was used to determine whether ARID1A is a target gene of miR-144-3p. The 3′UTR of the ARID1A sequence containing the predicted miR-144-3p binding sites and its mutant were cloned into the plasmid vector containing the firefly luciferase reporter gene. Subsequently, the constructed firefly luciferase vector (wild-type or mutated), Renilla luciferase vector and synthetic miR-144-3p mimic (or miR-Control) were co-transfected into HEK293 cells. Luciferase activity were measured using the dual luciferase assay system (Promega, Madison, WI, USA) according to the manufacturer’s instructions. The results are expressed as the ratio of firefly luciferase activity to the corresponding Renilla luciferase activity. All assays were performed in triplicate.

### miR-144-3p mimic and inhibitor transfection

The miR-144-3p mimic, miR-144-3p inhibitor, control of mimic and inhibitor were purchased from RiboBio Co., Ltd. (Guangzhou, China). The tenocytes were seeded onto cell culture plates at a density of 5000 cells per square centimeter 24 h before transfection. The cells were divided into five groups: (1) blank; (2) mimic negative control; (3) miR-144-3p mimic; (4) inhibitor negative control; (5) miR-144-3p inhibitor. After being cultured for 24 h, the cells were transfected with the miR-144-3p mimic or inhibitor at a concentration of 10 nM for 48 h using lipofectamine 3000 (Invitrogen, USA) according to the manufacturer’s instructions. Briefly, synthesized miRNA sequences and lipofectamine 3000 were mixed separately with Opti-MEM (Invitrogen) and then mixed together to form transfection complexes. All transfection experiments were performed in triplicate. Subsequently, RNA and protein were extracted to determine the changes of ARID1A expression.

### Statistical analysis

Data were presented as mean ± standard errors of the mean by using Prism 6.0 software (GraphPad Prism). The unpaired Student’s t test was employed to analyze difference between two groups. In the setting of multiple groups, one-way ANOVA with Dunnett’s multiple comparison test was conducted for in vitro experiments (comparison between each group with control group), and one-way ANOVA with Tukey’s multiple comparison test was conducted for in vivo experiments. A *p* value less than 0.05 was considered statistically significant.

## Results

### Characterization of TDSCs and TDSC-Exos

**Fig. 1:**
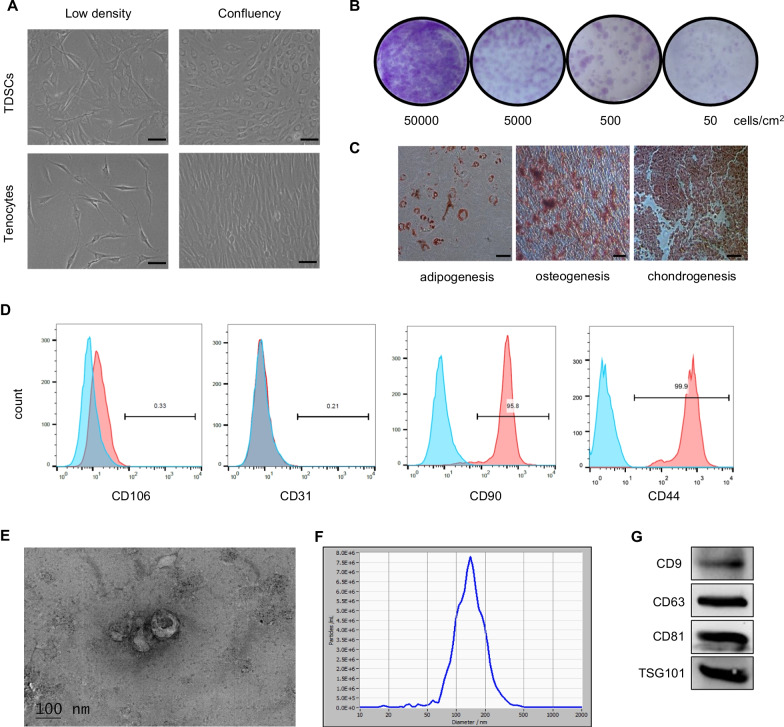
Characterization of TDSCs and TDSC-Exos. **A** Morphology of TDSCs and tenocytes. Scale bar: 50 μm. **B** Colony forming assay showed that TDSCs could form colony at a low cell density. **C** TDSCs exhibited multi-potential differentiation capacity for adipogenesis, osteogenesis and chondrogenesis. Scale bar: 50 μm. **D** Flow cytometric analysis of cell surface markers of TDSCs. Blue curves represent isotype controls, and red curves represent measured surface markers of TDSCs. **E** Morphology of TDSC-Exos observed by TEM. Scale bar: 100 nm. **F** The size distribution of TDSC-Exos peaks at 138.8 nm. **G** Exosomal markers (CD9, CD63, CD81 and TSG101) in TDSC-Exos were measured by western blotting

TDSCs and tenocytes were isolated from patellar tendon of rats. TDSCs were cobblestone-like after reaching confluence, and tenocytes were spindle-like in a confluent culture (Fig. 1A). Colony forming assay showed that TDSCs could form colony at a low cell density (Fig. 1B). TDSCs showed adipogenic, osteogenic and chondrogenic potential when cultured in the corresponding induction medium (Fig. 1C). Flow cytometric analysis revealed that TDSCs highly expressed the mesenchymal stem cell related markers CD90 (95.8%) and CD44 (99.0%) but did not express CD31 (0.2%) or CD106 (0.3%) (Fig. 1D), which was consistent with the previous study [25].

Exosomes derived from TDSCs were obtained by ultracentrifugation and identified using a TEM, Nanosight viewer and western blot analysis. The obtained microvesicles exhibited hollow spherical shape under TEM (Fig. 1E). Nanosight viewer showed that the diameter of the exosomes was about 139 nm (Fig. 1F). Western blot demonstrated the microvesicles were positive for exosome-related markers (CD9, CD63, CD81 and TSG101) (Fig. 1G). All these data suggested that the microvesicles isolated from TDSC-conditioned medium were exosomes.

### The effects of TDSC-Exos on the proliferation and function of tenocytes in vitro

**Fig. 2:**
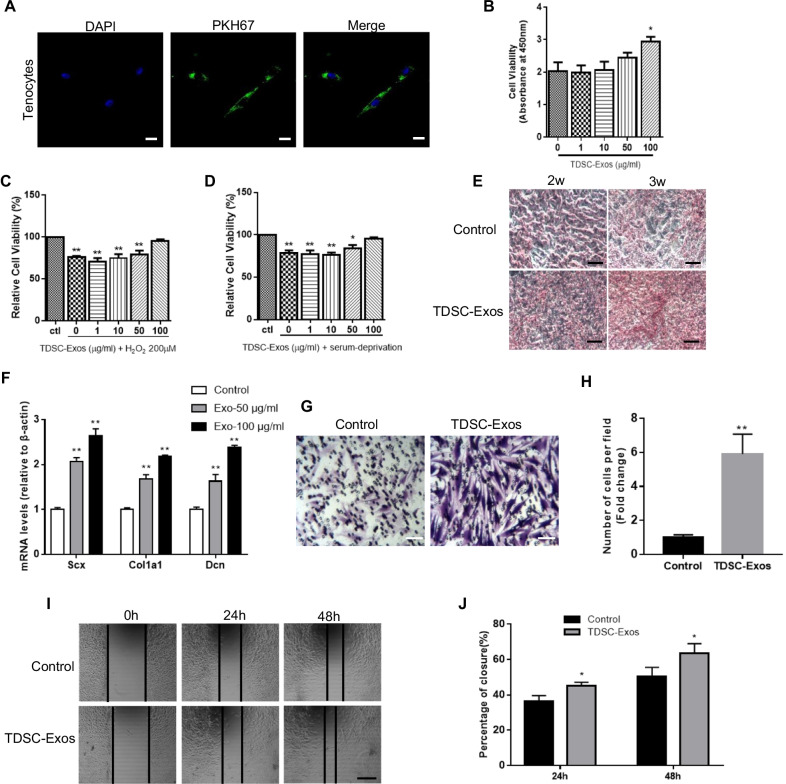
The effects of TDSC-Exos on tenocytes in vitro. **A** Tenocytes could absorb PKH67 (green)-labeled TDSC-Exos. The nuclei were stained with DAPI (blue). Scale bar: 20 μm. **B** Cell viability of tenocytes treated with TDSC-Exos at different concentrations (Bars: mean ± SE; *n* = 3; one-way ANOVA with Dunnett’s multiple comparison test, *p < 0.05 compared to control). **C**, **D** High concentration of exosomes could protect tenocytes from oxidative stress and serum deprivation (Bars: mean ± SE; *n* = 3; one-way ANOVA with Dunnett’s multiple comparison test, *p < 0.05 compared to control; **p < 0.01 compared to control). **E** Increased production of type I collagen was observed in tenocytes treated with TDSC-Exos by sirius red staining. Scale bar: 100 μm. **F** Tenocyte gene expression changes of Scx, Col1a1 and Dcn after treated with TDSC-Exos (Bars: mean ± SE; *n* = 3; one-way ANOVA with Dunnett’s multiple comparison test, **p < 0.01 compared to control). **G** Transwell assay of tenocytes treated with TDSC-Exos. Scale bar: 50 μm. **H** Quantitative analysis of the migrated cells in (**G**) (Bars: mean ± SE; *n* = 3; unpaired Student’s t test, **p < 0.01 compared to control). **I** TDSC-Exos promoted tenocyte migration in scratch wound healing assay. Scale bar: 250 μm. **J** Quantitative analysis about the percentage of closure in (**I**) (Bars: mean ± SE; *n* = 3; unpaired Student’s t test, *p < 0.05 compared to control)

Before we explore the effects of TDSC-Exos on tenocytes, the uptake of exosomes by tenocytes should be confirmed. Tenocytes were cultured in the medium with PKH67-labeled exosomes for 6 h. Internalized exosomes were observed in the perinuclear region of tenocytes using fluorescence microscopy (Fig. 2A), which indicated that tenocytes could absorb free TDSC-Exos in the medium.

Different concentrations of TDSC-Exos (1, 10, 50, 100 μg/mL) were used to treat tenocytes for 24 h, and CCK-8 assay was conducted to identify the proliferation of tenocytes. The data showed that high concentration of exosomes (100 μg/mL) promoted the proliferation of tenocytes (Fig. 2B). Subsequently, we cultured tenocytes in the medium with hydrogen peroxide or without serum to simulate the conditions of oxidative stress and starvation, respectively. Different concentrations of exosomes (1, 10, 50, 100 μg/mL) were added into the medium. The results revealed that high concentration of exosomes could protect tenocytes from oxidative stress and serum deprivation in vitro (Fig. 2C, D).

During the process of tendon repair, the ability of tenocytes to synthesize collagen is important. We treated tenocytes with TDSC-Exos for 10 days. Subsequently, sirius red staining was performed to evaluate the production of type I collagen. As a result, the increased production of type I collagen was observed in tenocytes treated with TDSC-Exos (Fig. 2E). Besides, qRT-PCR showed that TDSC-Exos treatment elevated the expression of tendon-specific markers (Scx, Col1a1 and Dcn) in tenocytes (Fig. 2F).

The migration of tenocytes to the injury site also represents a mechanism in the process of tendon repair. Tenocytes were cultured in the transwell plate with TDSC-Exos in the lower chamber for 24 h. The number of tenocytes migrating through the permeable membrane was almost six times as many as that of control group (Fig. 2G, H). Besides, we cultured tenocytes in the plate and made a scratch to produce a cell-free area. The width of scratch was narrower in the plates treated with TDSC-Exos than that in the control plates after 24 h and 48 h(Fig. 2I, J). These data suggested that TDSC-Exos could enhance the migration of tenocytes.

### The performance of pHA-TDSC-Exos scaffold

**Fig. 3:**
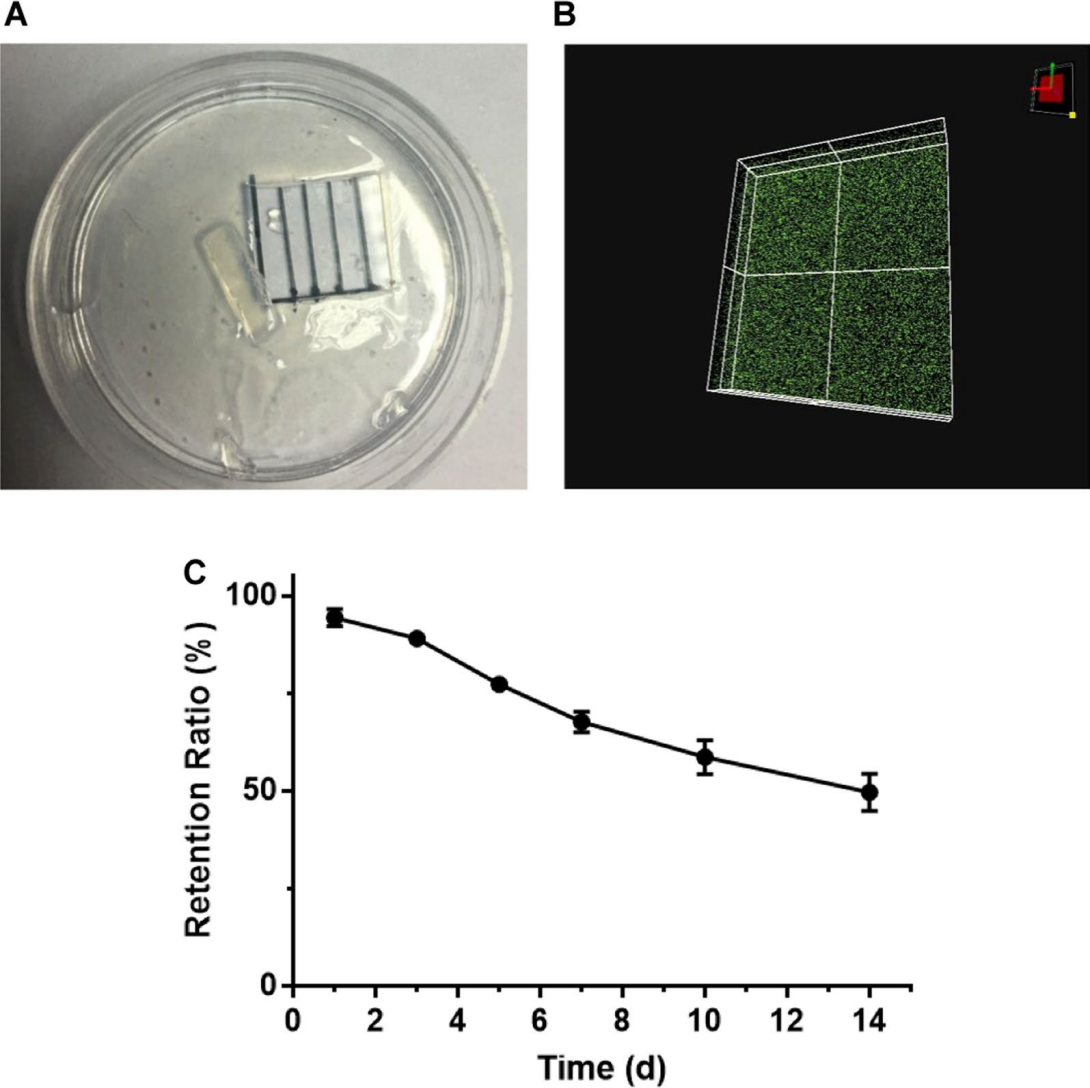
The performance of pHA-TDSC-Exos scaffold. **A** Gross view of pHA-TDSC-Exos scaffold. **B** The distribution of TDSC-Exos in the HA gel. **C** The exosomes retention ability of the HA gel. About 50% of the exosomes were still retained in the scaffold after 14 days (Bars: mean ± SE; *n* = 3)

PBS containing TDSC-Exos was mixed with p-HA lyophilized powder to produce the pHA-TDSC-Exos scaffold by irradiation (Fig. 3A). To determine the distribution of exosomes in the HA gel, we labeled exosomes with fluorescent dye CM-Dil. The uniform distribution of exosomes in the scaffold was observed using fluorescence microscopy (Fig. 3B). In order to explore the exosomes retention ability of the HA gel, Nanosight viewer was used to detect the exosomes released from the scaffold. About 50% of the exosomes were still retained in the scaffold after 14 days (Fig. 3C), which indicated that the scaffold could serve as an exosome sustained-release system.

### pHA-TDSC-Exos scaffold promoted tendon repair in the rat model

**Fig. 4:**
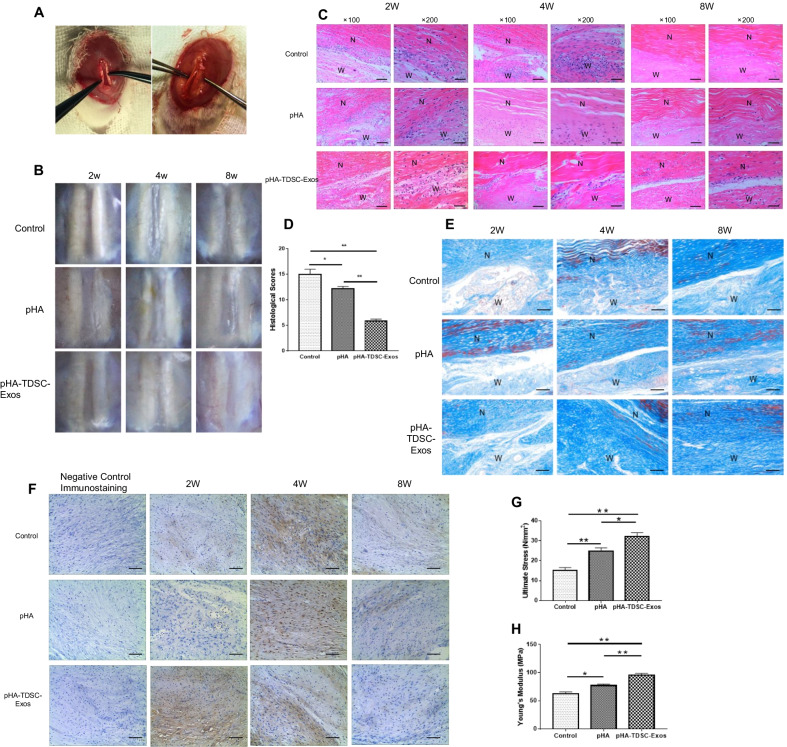
pHA-TDSC-Exos scaffold promoted the repair of tendon defects in the rat model. **A** The rat model of tendon defect was treated with pHA-TDSC-Exos scaffold. **B** Gross view of injured tendons treated with pHA or pHA-TDSC-Exos at 2, 4 and 8 weeks after surgery. **C** H&E staining of wound sections treated with pHA or pHA-TDSC-Exos at 2, 4 and 8 weeks after surgery. N: normal area; W: wound area. Scale bar: 200 μm (× 100); 100 μm (× 200). **D** Histological scores of tendons at 8 weeks after surgery (Bars: mean ± SE; *n* = 6; one-way ANOVA with Tukey’s multiple comparison test, *p < 0.05 and **p < 0.01). **E** Masson’s trichrome staining of wound sections treated with pHA or pHA-TDSC-Exos at 2, 4 and 8 weeks after surgery. N: normal area; W: wound area. Scale bar: 200 μm. **F** Immunohistochemistry of type III collagen during the process of tendon repair. Scale bar: 100 μm. **G** The ultimate stress of regenerated tissues treated with pHA or pHA-TDSC-Exos at 8 weeks after surgery (Bars: mean ± SE; *n* = 6; one-way ANOVA with Tukey’s multiple comparison test, *p < 0.05 and **p < 0.01); **H** The elastic modulus of regenerated tissues treated with pHA or pHA-TDSC-Exos at 8 weeks after surgery (Bars: mean ± SE; *n* = 6; one-way ANOVA with Tukey’s multiple comparison test, *p < 0.05 and **p < 0.01)

The central one-third of the patellar tendon was dissected to create the rat model of tendon defect. We used pHA or pHA-TDSC-Exos to fill the window gap in the patellar tendons (Fig. 4A). Rats, which did not receive the treatment of pHA or pHA-TDSC-Exos, served as control. We harvested the tendons to determine the wound healing outcomes at 2, 4 and 8 weeks postoperatively. The tendon defects in the control group remained obvious during the whole time period. The wound became much less visible with time in the group treated with pHA-TDSC-Exos (Fig. 4B).

The tendon tissues were then stained with H&E to conduct histological analysis (Fig. 4C). At 2 weeks after surgery, the cells in the window wound were less rounded and the cell alignment was better in the pHA-TDSC-Exos group compared with pHA and control groups. The cellularity reduced and extracellular matrix increased with time in all groups after 2 weeks postoperatively. At 4 weeks after surgery, more spindle-like cells and better fiber arrangement could be observed in the pHA-TDSC-Exos group than those in the other groups. At 8 weeks after surgery, the cells in the wound continued to reduce and the fiber was cluttered in the pHA and control groups. By comparison, the window wound in the pHA-TDSC-Exos group was very similar to the normal tendon tissue. According to the histological scoring, the wound healing outcomes in the pHA-TDSC-Exos group were significantly better than those in the pHA and control groups (Fig. 4D). The results indicated that pHA-TDSC-Exos could enhance tendon repair in the rat model.

Masson trichrome staining was employed to evaluate the regenerated collagen fibers in the window wound. The collagen increased with time in all groups, but better collagen fiber arrangement could be observed in the pHA-TDSC-Exos group compared to those in the pHA and control groups (Fig. 4E).

Type III collagen, which is mainly produced at the early stage of tendon healing, was assessed by using immunohistochemistry (Fig. 4F). Type III collagen was obviously observed at 2 weeks after surgery and then reduced with time in the pHA-TDSC-Exos group. In the pHA and control groups, type III collagen could be observed at 4 weeks after surgery and reduced significantly at 8 weeks after surgery. The data revealed that pHA-TDSC-Exos promoted the early repair of injured tendon.

At 8 weeks after surgery, the regenerated tendon tissue in the window wound was harvested to perform biomechanical testing. Ultimate stress and Young’s modulus were significantly increased in the pHA-TDSC-Exos group compared to those in the pHA and control groups (Fig. 4G, H). This indicated that pHA-TDSC-Exos facilitated the restoration of biomechanical property in the injured tendon.

### miRNA expression profiling in TDSC-Exos

**Fig. 5:**
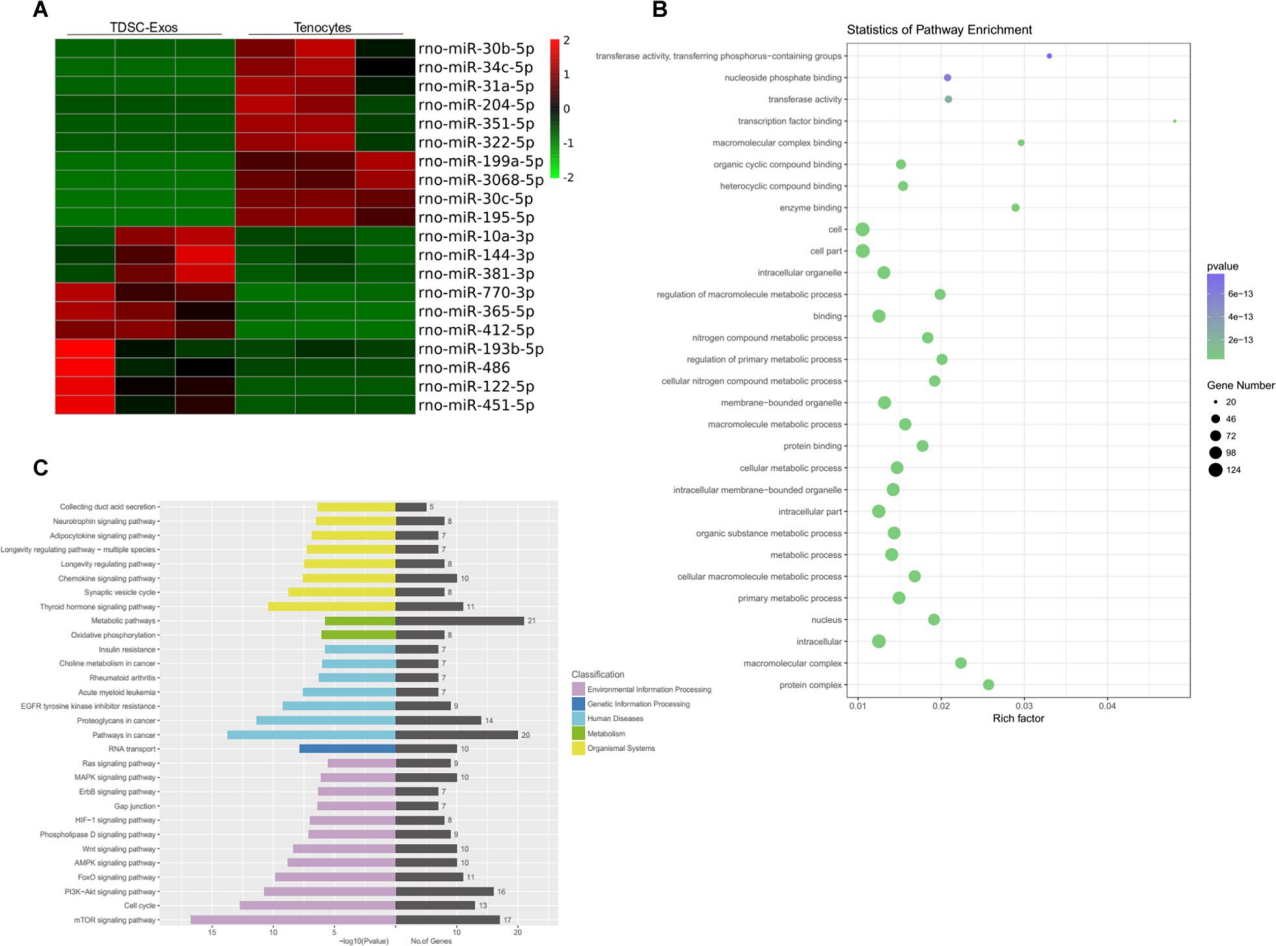
miRNA sequencing and bioinformatic analyses. **A** Heatmap of TDSC-Exo and tenocyte miRNAs. The red dots indicate up-regulated miRNAs and the green dots indicate down-regulated miRNAs. **B** GO analysis revealed the most related biological processes of the top 10 highly expressed miRNAs in TDSC-Exos. **C** KEGG analysis determined the most related signaling pathways of the top 10 highly expressed miRNAs in TDSC-Exos

Increasing number of studies have indicated that exosomal miRNAs played an important role in the tissue repair. To explore the role of miRNAs in the process of TDSC-Exos-regulated tendon repair, we performed RNA sequencing to detect the miRNA expression profiles of TDSC-Exos and tenocytes. A total of 20 miRNAs were found to be expressed significantly higher in TDSC-Exos compared to tenocytes (fold change > 1.0; *p* < 0.05) (Table 1). Among them, the top 10 enriched miRNAs in TDSC-Exos are miR-412-5p, miR-122-5p, miR-451-5p, miR-770-3p, miR-365-5p, miR-486, miR-10a-3p, miR-144-3p, miR-193b-5p and miR-381-3p (Fig. 5A).

**Table 1:** Enriched miRNAs in TDSC-Exos compared to tenocytes

miRNA_ID	Up/down	log2 (fold change)	*p* value	Significance
rno-miR-412-5p	Up	9.3703	1.19E−30	**
rno-miR-122-5p	Up	8.0753	9.44E−29	**
rno-miR-451-5p	Up	6.1553	4.58E−21	**
rno-miR-770-3p	Up	5.2319	1.57E−20	**
rno-miR-365-5p	Up	4.7898	1.51E−14	**
rno-miR-486	Up	3.7645	1.02E−15	**
rno-miR-10a-3p	Up	3.6929	6.53E−11	**
rno-miR-144-3p	Up	3.2553	3.21E−10	**
rno-miR-193b-5p	Up	2.8531	1.90E−08	**
rno-miR-381-3p	Up	2.7576	4.87E−11	**
rno-miR-760-3p	Up	2.5723	1.21E−17	**
rno-miR-139-3p	Up	2.3287	1.15E−05	**
rno-miR-375-3p	Up	2.2227	9.89E−06	**
rno-miR-204-3p	Up	1.9315	1.25E−11	**
rno-miR-409a-3p	Up	1.8591	1.52E−16	**
rno-miR-323-3p	Up	1.7207	2.16E−12	**
rno-miR-142-5p	Up	1.4116	3.93E−08	**
rno-miR-144-5p	Up	1.3945	0.007376	**
rno-miR-134-5p	Up	1.1823	2.83E−17	**
rno-miR-423-5p	Up	1.1527	4.47E−12	**

**p < 0.01 compared to tenocytes

Candidate target genes of the top 10 enriched miRNAs were predicted by four databases (TargetScan, miRanda, miRWalk, and miRTarBase), and then STRING online database was employed to identify those PPIs with a combined score > 0.9. A total of 153 genes were included in the miRNA-gene networks (Additional file 1). We also obtained the PPI network to explore the interactions between these target genes (Additional file 2). Cdk1 and Mtor were the genes interacting with the largest number of other genes, followed by Atp6v1a, Atp6v1b1, Atp6v1d, Atp6v1h, Atp6v1g1, Atp6v1g3, Med13, Med14 and Smarcb1 (Additional file 3).

Subsequently, GO terms and KEGG pathway enrichment analyses were performed to explore the most related processes and signaling pathways. According to the GO analysis (Fig. 5B), the top 10 enriched miRNAs were most associated with transcription factor binding in terms of molecular function, followed by transferase activity of transferring phosphorus—containing groups, macromolecular complex binding and enzyme binding. The most involved biological process was the regulation of primary metabolic process. Protein complex and macromolecular complex were the most enriched cellular components. The KEGG analysis revealed that these miRNAs were mainly involved in the metabolic pathways. In the respect of environmental information processing, mTOR signaling pathway, PI3K-Akt signaling pathway and cell cycle were the most related pathways (Fig. 5C).

### miR-144-3p was a key component in TDSC-Exos-regulated tendon repair

**Fig. 6:**
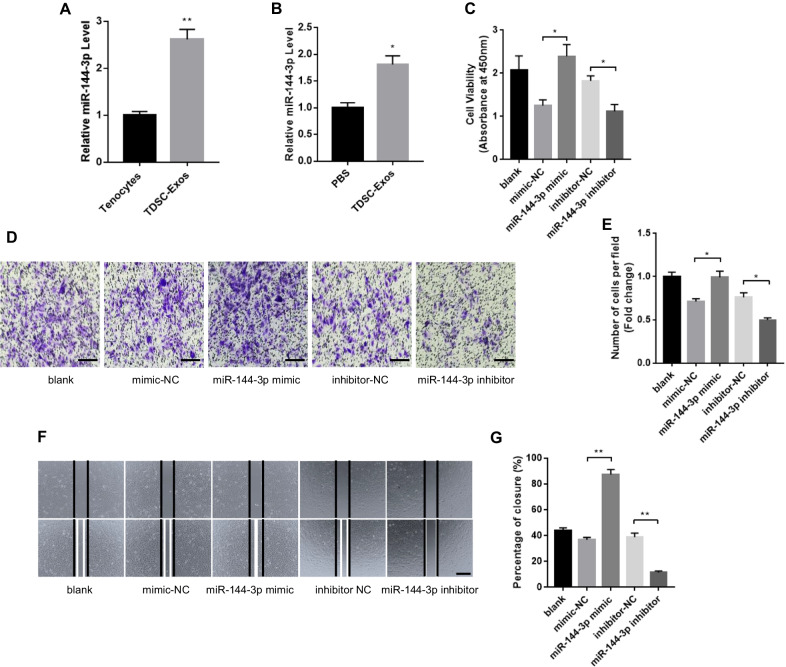
miR-144-3p from TDSC-Exos promoted tenocytes proliferation and migration. **A** The higher expression of miR-144-3p in TDSC-Exos compared with tenocytes was confirmed by qRT-PCR (Bars: mean ± SE; *n* = 3; unpaired Student’s t test, **p < 0.01). **B** Compared with PBS treatment, TDSC-Exos treatment increased miR-144-3p expression in tenocytes (Bars: mean ± SE; *n* = 3; unpaired Student’s t test, *p < 0.05). **C** miR-144-3p mimic significantly increased tenocytes proliferation, while miR-144-3p inhibitor suppressed tenocytes proliferation (Bars: mean ± SE; *n* = 3; unpaired Student’s t test: mimic-NC vs. miR-144-3p mimic; inhibitor-NC vs. miR-144-3p inhibitor, *p < 0.05). **D** Transwell assay showed that miR-144-3p mimic promoted tenocytes migration and miR-144-3p inhibitor suppressed tenocytes migration. Scale bar: 100 μm. **E** Quantitative analysis of the migrated cells in (**D**) (Bars: mean ± SE; *n* = 3; unpaired Student’s t test: mimic-NC vs. miR-144-3p mimic; inhibitor-NC vs. miR-144-3p inhibitor, *p < 0.05). **F** miR-144-3p mimic promoted tenocytes migration and miR-144-3p inhibitor suppressed tenocytes migration in scratch wound healing assay. Scale bar: 500 μm. **G** Quantitative analysis about the percentage of closure in (**F**) (Bars: mean ± SE; *n* = 3; unpaired Student’s t test: mimic-NC vs. miR-144-3p mimic; inhibitor-NC vs. miR-144-3p inhibitor, **p < 0.01)

We further focused on miR-144-3p, which has proven to enhance cell proliferation and migration by previous studies [26, 27]. The up-regulated expression of miR-144-3p in TDSC-Exos was further confirmed by qRT-PCR analysis (Fig. 6A). To determine whether exosomal miR-144-3p could be transferred to tenocytes, we evaluated miR-144-3p levels in tenocytes treated with TDSC-Exos or PBS after 24 h. An increase of the cellular level of miR-144-3p was observed in tenocytes receiving TDSC-Exos treatment (Fig. 6B), indicating that TDSC-Exos delivered miR-144-3p to tenocytes.

### miR-144-3p promoted tenocyte proliferation and migration

To verify whether miR-144-3p could regulate tendon repair, the effects of miR-144-3p on tenocyte proliferation and migration were evaluated. miR-144-3p mimic and inhibitor were transfected into tenocytes for gain- and loss-of function analyses. The CCK-8 assay was conducted to explore the effects of miR-144-3p mimic and inhibitor on tenocyte proliferation. It was showed that miR-144-3p mimic notably promoted tenocyte proliferation. In contrast, miR-144-3p inhibitor suppressed the proliferation of tenocytes (Fig. 6C). Transwell and scratch wound assay were used to assess the migration of tenocytes after treatment by miR-144-3p mimic or inhibitor. According to the results, miR-144-3p mimic significantly promoted the migratory ability of tenocytes, while miR-144-3p inhibitor attenuated tenocyte migration (Fig. 6D–G).

### miR-144-3p promoted tendon repair via targeting ARID1A in tenocytes

**Fig. 7:**
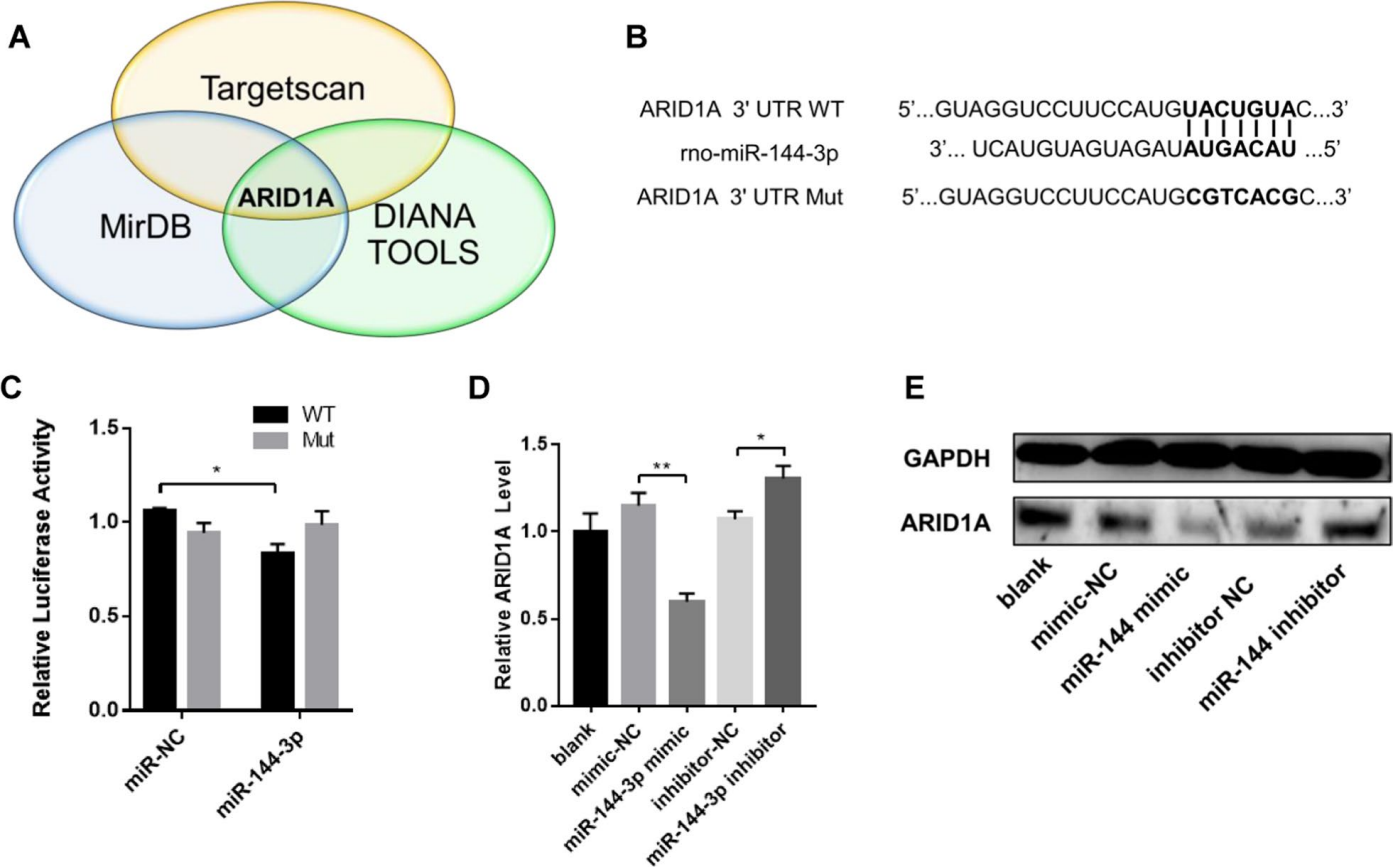
miR-144-3p promoted tendon repair via targeting ARID1A in tenocytes. **A** ARID1A was predicted as a potential target of miR-144-3p by all three bioinformatic programs (TargetScan, miRDB and DIANA TOOLS). **B** Plasmid vectors of ARID1A 3′UTR and its mutation. **C** Dual-luciferase reporter assay showed that miR-144-3p targeted ARID1A 3′UTR but not its mutation (Bars: mean ± SE; *n* = 3; unpaired Student’s t test: miR-NC vs. miR-144-3p in wild type groups; miR-NC vs. miR-144-3p in mutant groups, *p < 0.05). **D**, **E** miR-144-3p mimic suppressed ARID1A mRNA and protein expression in tenocytes, while miR-144-3p inhibitor removed the suppression effects (Bars: mean ± SE; *n* = 3; unpaired Student’s t test: mimic-NC vs. miR-144-3p mimic; inhibitor-NC vs. miR-144-3p inhibitor, *p < 0.05 and **p < 0.01)

ARID1A was predicted as a potential target of miR-144-3p by all three bioinformatic programs (TargetScan, miRDB and DIANA TOOLS) (Fig. 7A). The downregulation of ARID1A has proven to enhance mammalian regeneration and promote tissue repair after injury [28]. Therefore, we speculated that miR-144-3p from TDSC-Exos performed its function in tendon repair by downregulating ARID1A in tenocytes. To confirm whether ARID1A was the direct target of miR-144-3p, we conducted the dual-luciferase reporter assay (Fig. 7B). miR-144-3p mimic suppressed the firefly luciferase activity in the ARID1A 3′-UTR wild-type group but not decreased that in the mutation group, which revealed the direct binding of miR-144-3p to the 3′-UTR of ARID1A (Fig. 7C). In order to investigate whether miR-144-3p could downregulate ARID1A in tenocytes, miR-144-3p mimic and inhibitor were transfected into tenocytes. Real-time qPCR showed that miR-144-3p mimic suppressed the expression of ARID1A and miR-144-3p inhibitor increased the expression of ARID1A (Fig. 7D). Consistently, western blot analysis showed that miR-144-3p mimic suppressed ARID1A protein expression and miR-144-3p inhibitor removed the suppression effects (Fig. 7E). These results suggested that miR-144-3p from TDSC-Exos played an important role in tendon repair by targeting ARID1A in tenocytes.

## Discussion

In this study, we revealed that TDSC-Exos promoted proliferation, migration, type I collagen production and tendon-specific markers expression in tenocytes, and also protected tenocytes from oxidative stress and serum deprivation. Subsequently, we established a pHA-TDSC-Exos scaffold and proved that this scaffold could facilitate tendon repair in the rat model. In order to explore the underlying mechanism, we compared the miRNA expression profiles between TDSC-Exos and tenocytes. Then, we focused on miR-144-3p, which was enriched in TDSC-Exos. We found that exosomal-transferred miR-144-3p could promote tenocyte proliferation and migration via targeting ARID1A.

Exosomes derived from various cellular sources have shown to promote musculoskeletal tissues repair and regeneration. Exosomes secreted by BMSCs promote myogenesis in vitro and muscle regeneration in a mouse model of muscle injury [29]. Exosomes released during the differentiation of myoblasts into myotubes can induce the myogenesis of ADSCs and improve myofiber regeneration at the injury site [30]. A scaffold combining exosomes derived from mesenchymal stem cells (MSCs) and tricalcium phosphate can repair bone defects by activating the PI3K/Akt signaling pathway [31]. Besides, several studies have indicated that exosomes could be applied to treat osteoporosis. BMSC-secreted exosomes improve osteoporosis through promoting osteoblast proliferation and inhibiting cell apoptosis [32, 33]. Furthermore, endothelial cell-secreted exosomes have shown to be more efficient in bone targeting than BMSC-secreted exosomes, and they can alleviate osteoporosis by inhibiting osteoclast activity [34]. Emerging evidence suggests that exosomes can also improve cartilage regeneration and alleviate osteoarthritis. Exosomes derived from embryonic MSCs mediate cartilage repair through enhancing chondrocyte proliferation, increasing type II collagen synthesis, attenuating degradation, and regulating immune reactivity [35, 36]. Exosomes secreted from infrapatellar fat pad MSCs proved to maintain cartilage homeostasis through exosomal miR-100-5p-regulated inhibition of mTOR-autophagy pathway [37]. Exosomes released by miR-140-5p-overexpressing synovial MSCs promote cartilage regeneration and prevent osteoarthritis in a rat model, which suggests the promising role of exosomes from modified cells in tissue repair [14].

Since exosomes can be secreted by various cells, the choice of the cell type should be considered before the study. Compared with other types of cells, TDSCs represent a better performance in clonogenicity, proliferation, and tenogenic differentiation potential [9], which suggests that TDSCs may be an ideal cellular source for exosomes obtaining to treat tendon injury. Several studies regarding the effects of TDSC-Exos on tendon repair have been published recently. TDSC-Exos could balance the synthesis and degradation of tendon extracellular matrix and promote the tenogenesis of resident TDSCs [38]. TDSC-Exos were also reported to suppress the inflammation and apoptosis in the process of tendon healing in the Achilles tendon injury models [39]. Besides, TDSC-Exos could promote the proliferation and migration of TDSCs by activating the TGF β signaling pathway [40]. In this study, we mainly explored the effects of TDSC-Exos on tenocytes in vitro. Since there was no study regarding the role of exosomal miRNA in tendon healing, we performed RNA sequencing to detect the miRNA expression profiles of TDSC-Exos and determined the role of exosomal miR-144-3p in tendon repair.

Tenocytes are the most abundant cell type in tendon tissue, and play a critical role in tendon repair. Therefore, we first explored the effects of TDSC-Exos on tenocytes in vitro. In order to determine the therapeutic effects of TDSC-Exos in vivo, we used a pHA-TDSC-Exos scaffold to treat rats with patellar tendon defects. The scaffold served as a sustained release system to avoid the iatrogenic injury caused by repeated injection. We found that TDSC-Exos enhanced proliferation and migration of tenocytes in vitro, which may partially explain the efficient tendon repair in the rat model. At the injury site, tenocytes are exposured to an environment of oxidative stress and inadequate nutrition. In order to determine the cytoprotective ability of TDSC-Exos, we cultured tenocytes in the corresponding medium to mimic the injured condition. The results indicated that TDSC-Exos could protect tenocytes from oxidative stress and serum deprivation. The hierarchical collagen structure is essential to the function of tendon. In this study, TDSC-Exos could increase tenocyte collagen production in vitro, and rats treated with TDSC-Exos had better fiber arrangement at the injury site. During the process of tendon healing, type III collagen is synthesized largely at the early stage and then gradually replaced by type I collagen. In our study, injured tendon treated with TDSC-Exos exhibited the earliest expression of type III collagen, which indicated that TDSC-Exos promoted early healing of tendon. Besides, the injured tendons in the rats treated with TDSC-Exos had better performance in biomechanical testing. In conclusion, we proved that TDSC-Exos could enhance tendon repair through in vitro and in vivo studies. However, we only conducted histological analyses and biomechanical tests to evaluate the reparative effects of TDSC-Exos in the rat model. Owing to a lack of evaluation of rat behavior associated with tendon injury, we were unable to assess the clinical outcomes in vivo. Therefore, methods such as weight-bearing, mechanical sensitivity, gait analysis and motion analysis are warranted in further studies to evaluate the behavior of animal models.

Exosomal miRNAs have proven to play an important role in the process of tissue repair and regeneration, such as promoting angiogenesis [41, 42], mediating inflammation [43] and regulating cellular function [42]. Therefore, we attempted to explain the therapeutic effects of TDSC-Exos on tendon injury by exploring the role of miRNAs in this study. We performed RNA sequencing to explore the miRNA expression profiles of TDSC-Exos and tenocytes. Among the enriched miRNAs in TDSC-exos compared with tenocytes, we focused on miR-144-3p. The function of miR-144-3p has been studied in different diseases, suggesting that overexpression of miR-144-3p could promote cell proliferation and migration [26, 27, 44]. According to the results of gain- and loss-of function analyses in the present study, miR-144-3p could enhance the proliferation and migration of tenocytes, which may play a role in the process of tendon repair. In order to further explore the underlying mechanism, we used bioinformatics programs to predict the potential target genes. ARID1A was predicted by all three bioinformatic programs, and was then proved to be the target of miR-144-3p by dual-luciferase reporter assay, which is consistent with a previous study [27].

ARID1A is a key component of switch/sucrose nonfermentable (SWI/SNF) ATP-dependent chromatin-remodeling complex, which plays a critical role in the modulation of cell cycle [45]. The SWI/SNF chromatin remodeling complexes support terminal differentiation and suppressed cell-cycle re-entry, which indicates that it functions as a regeneration suppressor. The downregulation of ARID1A disrupts SWI/SNF targeting and subsequent chromatin remodeling via restricting transcriptional access by C/EBPα and E2F4 [28]. ARID1A is physiologically repressed during liver regeneration and wound healing, and deletion of ARID1A enhances tissue repair without excessive overgrowth [28]. The conditional ablation of ARID1A has also proven to promote the epithelial proliferation in uterus [46]. In a previous study regarding clear cell renal cell carcinoma [27], ARID1A was identified as a direct target gene of miR-144-3p. They also found that miR-144-3p overexpression could promote cell proliferation and migration via targeting ARID1A, which is consistent with the present study.

It is noteworthy that miR-144-3p is only one of the potential mechanisms explaining the therapeutic effects of TDSC-Exos. Other miRNAs may also play an important role in tendon repair, and bioinformatic analysis in the present study has also revealed that they could repair tendon injury via different processes and pathways. GO analysis showed that enriched miRNAs mainly involved in the processes of binding and metabolism, indicating that miRNAs had important roles in regulating recipient cells. According to KEGG analysis, many pathways may be involved in the TDSC-Exos-regulated tendon repair. The PI3K/AKT/mTOR pathway is important in regulating cell cycle, and closely related to proliferation and regeneration. In the tendon tissues, mTOR signaling pathway has been reported to participate in the senescence and tendinopathy [47, 48]. Activation of AKT-mTOR signaling pathway can promote type I collagen production and tenogenesis of MSC [48]. Besides, mTOR signaling pathway regulates the proliferation and differentiation of TDSCs, indicating its potential role in tendon repair by stimulating endogenous stem/progenitor cells [49]. Besides, the role of FoxO pathway in regulating cell fate has been widely recognized. In tenocytes, FOXO3a activation can promote either cell survival or apoptosis in diverse culture circumstances [50]. The AMPK pathway is involved in the maintenance of tendon homeostasis by regulating the function of tenocytes [51]. The Wnt/β-catenin signaling has been demonstrated to suppress tenogenic gene expressions in tenocytes [52]. The MAPK signaling pathway participates in various processes of tendon healing, including cell proliferation, collagen synthesis, tenogenesis, tendon growth and remodeling [53, 54]. Furthermore, the other contents of TDSC-Exos, such as proteins and mRNAs, may also promote tendon repair via a variety of methods. Therefore, the therapeutic effects of TDSC-Exos should be recognized as the result of a combination of different exosomal contents.

## Conclusions

In conclusion, the present study suggests that exosomes derived from TDSCs can promote the repair of injured tendon, and exosomal-transferred miR-144-3p enhances the proliferation and migration of tenocytes via targeting ARID1A. Our findings provide a new potential strategy to treat tendon injury.

## Supplementary Information

**Additional file 1.** The network of top 10 enriched miRNAs in TDSC-Exos and target genes. A total of 153 genes were predicted as the targets of the top 10 enriched miRNAs in TDSC-Exos by four databases (TargetScan, miRanda, miRWalk, and miRTarBase).

**Additional file 2.** The protein–protein interaction (PPI) network of target genes. The STRING online database was used to identify those PPIs with a combined score more than 0.9. The PPI network was obtained to exhibit the interactions between these target genes.

**Additional file 3.** The genes interacting with the largest number of other genes. Cdk1 and Mtor were the genes interacting with the largest number of other genes, followed by Atp6v1a, Atp6v1b1, Atp6v1d, Atp6v1h, Atp6v1g1, Atp6v1g3, Med13, Med14 and Smarcb1.

## Data Availability

The datasets used and/or analyzed during the current study are available from the corresponding author on reasonable request.

## Abbreviations

ADSCs: Adipose-derived stem cells
Akt: Protein kinase B
AMPK: AMP-activated protein kinase
ARID1A: AT-rich interaction domain 1A
BCA: Bicinchoninic acid
BMSCs: Bone marrow mesenchymal stem cells
CCK-8: Cell counting kit-8
C/EBPα: CCAAT/enhancer binding protein α
DAPI: 4′,6-Diamidino-2-phenylindole
Dcn: Decorin
ESCs: Embryonic stem cells
E2F4: E2F transcription factor 4
FBS: Fetal bovine serum
FoxO: Forkhead box class O
GO: Gene ontology
H&E: Haemotoxylin and eosin
KEGG: Kyoto Encyclopedia of Genes and Genomes
LG-DMEM: Low glucose Dulbecco’s Modified Eagle Medium
MAPK: Mitogen-activated protein kinase
miRNA: MicroRNA
mRNA: Messenger RNA
MSCs: Mesenchymal stem cells
mTOR: Mammalian target of rapamycin
PCR: Polymerase chain reaction
PBS: Phosphate-buffered saline solution
PI3K: Phosphatidylinositol-3-kinase
PPI: Protein-protein interaction
P0: Passage 0
p-HA: Photopolymerizable hyaluronic acid
pHA-TDSC-Exos: Photopolymerizable hyaluronic acid loaded with TDSC-Exos
qRT-PCR: Real-time quantitative PCR
Scx: Scleraxis
SD: Sprague-Dawley
SWI/SNF: Switch/sucrose nonfermentable
TDSCs: Tendon derived stem cells
TDSC-Exos: Exosomes from tendon derived stem cells
TEM: Transmission electron microscopy
TSG101: Tumour susceptibility gene 101
3′UTR: The three prime untranslated region
