# Supplementary figures and images for: Exosomes from tendon derived stem cells promote tendon repair through miR-144-3p-regulated tenocyte proliferation and migration
Source: Stem Cell Res Ther. 2022 Feb 23;13:80. doi: 10.1186/s13287-022-02723-4 (PMC8867681; doi:10.1186/s13287-022-02723-4)

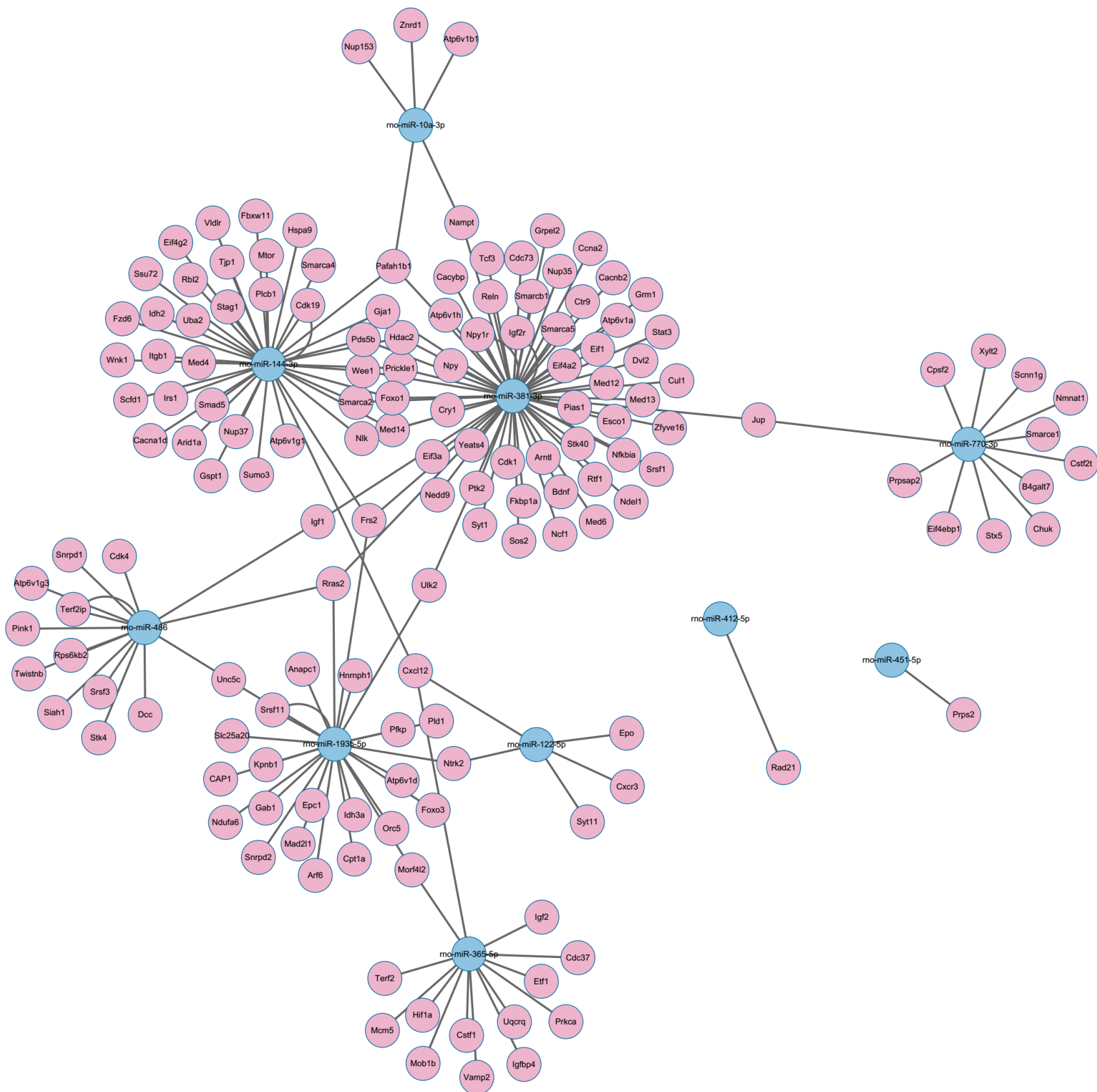

Supplement: Supplementary file 1 — Additional file 1. The network of top 10 enriched miRNAs in TDSC-Exos and target genes. A total of 153 genes were predicted as the targets of the top 10 enriched miRNAs in TDSC-Exos by four databases (TargetScan, miRanda, miRWalk, and miRTarBase). [file 13287_2022_2723_MOESM1_ESM.pdf]

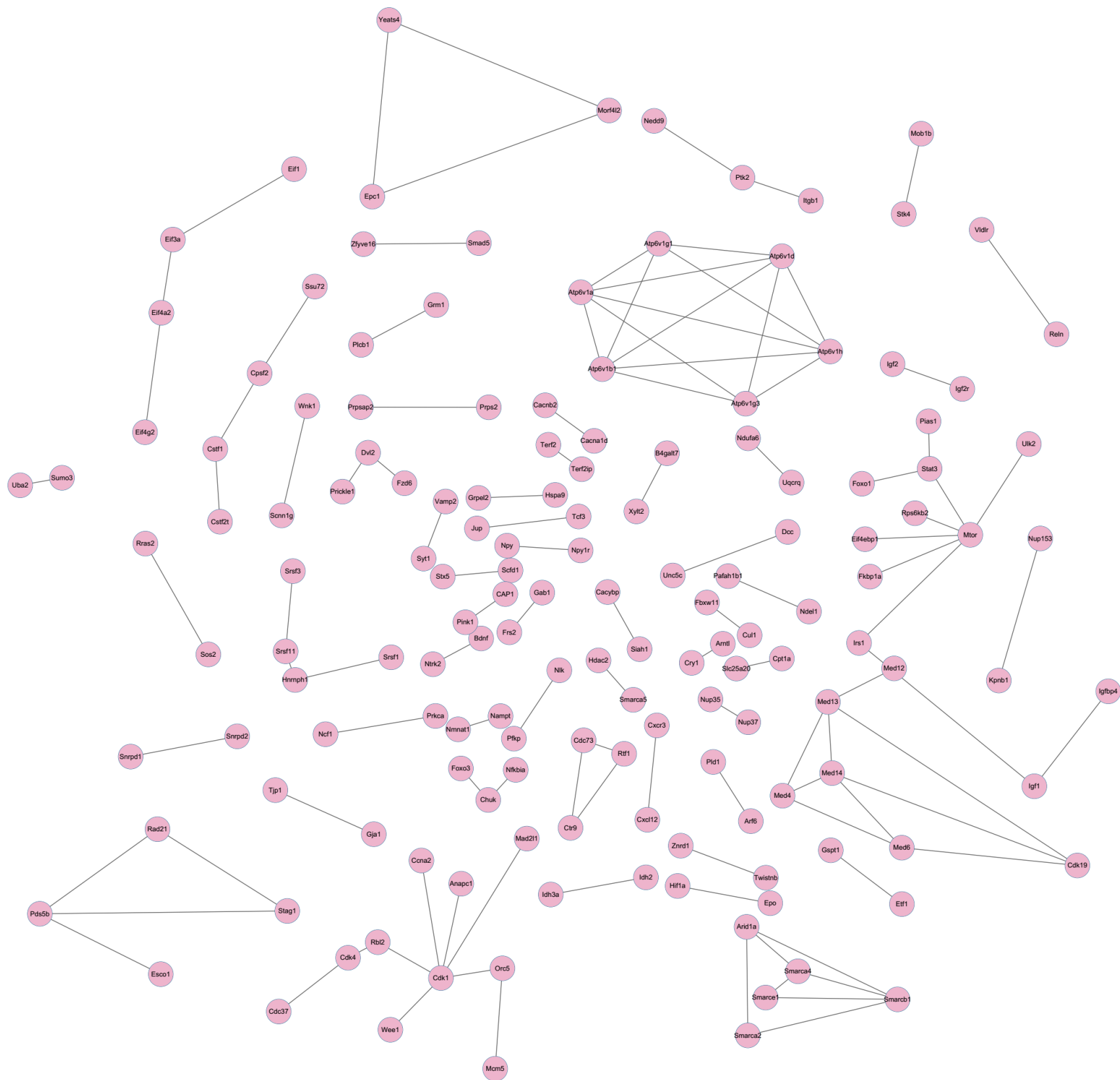

Supplement: Supplementary file 2 — Additional file 2. The protein–protein interaction (PPI) network of target genes. The STRING online database was used to identify those PPIs with a combined score more than 0.9. The PPI network was obtained to exhibit the interactions between these target genes. [file 13287_2022_2723_MOESM2_ESM.pdf]

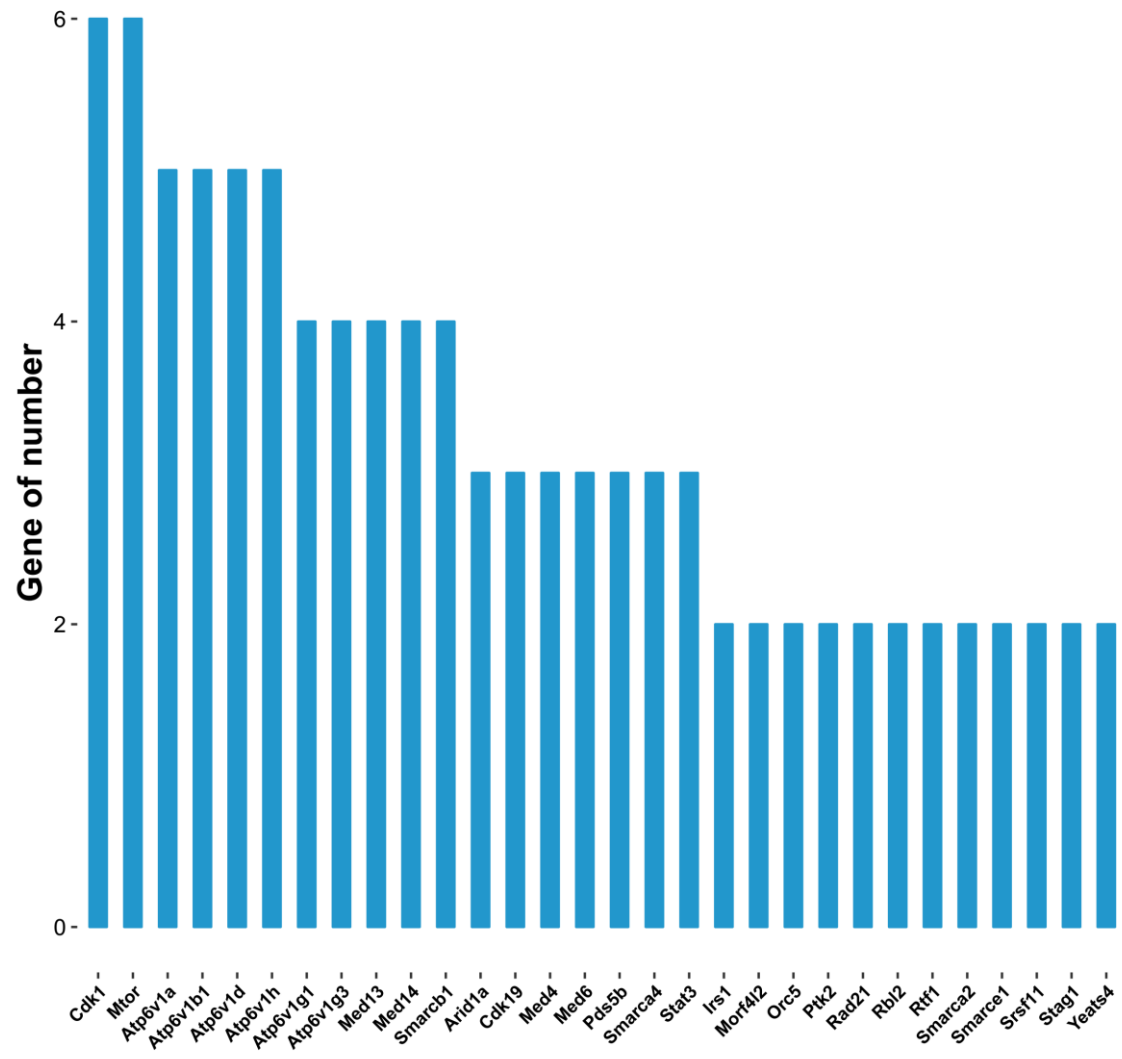

Supplement: Supplementary file 3 — Additional file 3. The genes interacting with the largest number of other genes. Cdk1 and Mtor were the genes interacting with the largest number of other genes, followed by Atp6v1a, Atp6v1b1, Atp6v1d, Atp6v1h, Atp6v1g1, Atp6v1g3, Med13, Med14 and Smarcb1. [file 13287_2022_2723_MOESM3_ESM.pdf]
